# Post-traumatic Stress Symptoms, Distress, and Optimism in Mexican Colorectal Cancer Patients

**DOI:** 10.11621/pir.2022.0408

**Published:** 2022-12-30

**Authors:** Jessica Noemí Acevedo-Ibarra, Dehisy Marisol Juárez-García, Absalón Espinoza-Velazco, Sergio Buenaventura-Cisneros, Arnoldo Téllez

**Affiliations:** a Universidad del Valle de México, Campus Cumbres, Departamento de Ciencias de la Salud, Monterrey, Nuevo León, México.; b UMAE Hospital de Especialidades No. 25, Centro Médico Nacional del Noreste, Instituto Mexicano del Seguro Social, Departamento de Oncología, Monterrey, Nuevo León, México; c Universidad Autónoma de Nuevo León, Facultad de Psicología y Centro de Investigación y Desarrollo en Ciencias de la Salud, Monterrey, Nuevo León, México

**Keywords:** Cancer, colorectal cancer, distress, post-traumatic stress, optimism

## Abstract

**Background:**

The diagnosis and treatment of colorectal cancer are considered highly stressful and potentially traumatic events that can generate post-traumatic stress symptoms and distress among patients.

**Objective:**

This study assessed levels of post-traumatic stress symptoms, distress, and optimism, as well as differences between these conditions relative to sociodemographic and medical variables, in Mexican patients with colorectal cancer.

**Design:**

A cross-sectional descriptive study design was employed, in which 192 colorectal cancer patients over the age of 18 years participated. They filled out the following questionnaires in person: a sociodemographic and medical data questionnaire; the Event Impact Scale-Revised (EIE-R); the Hospital Anxiety and Depression Scale (HADS); and the Life Orientation Test (LOT-R).

**Results:**

The results showed that 32.3% of the patients reported post-traumatic stress symptomatology, and 21.4% reported distress. Post-traumatic stress symptoms and distress varied according to age and monthly income. Significant differences between the sexes were also observed in the levels of post-traumatic stress symptoms and distress. Post-traumatic stress was positively and significantly related to distress, and negatively and significantly to optimism.

**Conclusion:**

Based on these data, we concluded that a significant percentage of colorectal cancer patients present high levels of post-traumatic stress symptoms as well as distress, and that these levels may vary according to sociodemographic and medical characteristics.

## Introduction

Colorectal cancer is considered the third most frequent type of cancer in Mexico, and ranks first in mortality (Organización Panamericana de la Salud [OPS], 2020). Evidence suggests that a substantial proportion of people with this form of cancer may experience their diagnosis and treatment as traumatic (Cordova et al., 2017).

Some studies have examined the prevalence of post-traumatic stress symptoms in adult cancer patients 6.5 months after treatment; the estimates range from 5% to 19%. In total, 10% of patients have reported such symptoms during the first six months after diagnosis and obtained a score above the clinical limit for probable post-traumatic stress disorder (Kangas et al., 2002; Schuster et al., 2014). It has been observed that patients with colorectal cancer with a diagnosis more than one year old, present greater post-traumatic stress, intrusive thoughts, and hypervigilance, compared to patients with a diagnosis less than six months old, or between six months and one year. Post-traumatic stress has also been reported to be a predictor of distress and intestinal pain (Pereira et al., 2012).

Post-traumatic stress symptoms are strongly associated with anxiety and distress, and a negative association with physical quality of life is also observed in the cancer population (Shand et al., 2015). Similarly, psychological distress has been negatively associated with health-related quality of life, independent of disease parameters, physical and mental indicators, and social relationships (Hyphantis et al., 2011; Paika et al., 2010).

In patients with colorectal cancer, having a stoma and low optimism are risk factors for lower quality of life and greater distress (Meng et al., 2012). In addition, having a stoma has a negative impact on a patient’s body image, which is a strong predictor of initial levels of anxiety, depression, and distress (Sharpe et al., 2011). For its part, optimism shows a positive association with a positive emotional quality of life (Acevedo-Ibarra et al., 2021). Higher optimism is associated with better global health-related quality of life and better physical, role, emotional, cognitive, and social functioning in colorectal cancer patients with sensory peripheral neuropathy in their one-year follow-up after diagnosis (Trompetter et al., 2022). In this way, optimism favors adaptation to the disease and acts as a protective factor by reducing patients’ vulnerability to other types of emotional and physical illnesses associated with cancer (Mera & Ortiz, 2012).

Currently, there is little information on the psychological alterations in Mexican patients with colorectal cancer. There are only two studies; one examining quality of life, which found that patients presented low emotional functioning (Balderas-Peña et al., 2011), and the other reviewing the psychosocial adaptation of the patient, which found that both the clinical characteristics of cancer and sociodemographic factors influence adaptation. That study, however, implied that there might be other factors that influence disease adaptation (Alvarado-Aguilar et al., 2011). Previous studies have focused on measuring quality of life and sociodemographic and medical aspects (Alvarado-Aguilar et al., 2011; Balderas-Peña et al., 2011).

Our study thus aimed to further advance the literature by providing theoretical and empirical scientific evidence on psychological variables such as symptoms of post-traumatic stress, distress, and optimism in patients with colorectal cancer during the disease, in order to identify those who need psychological care, and design more personalized support care interventions (Russell et al., 2015). Based on the above, our objective was to evaluate the levels and relationships of symptoms of post-traumatic stress, distress, and optimism, as well as to examine differences relative to sociodemographic and medical characteristics in Mexican patients with colorectal cancer. We sought to test the hypothesis that there is a correlation between symptoms of post-traumatic stress, distress, and optimism, as well as examine differences based on sociodemographic and medical characteristics in Mexican patients with colorectal cancer.

## Methods

### Participants

A descriptive cross-sectional design was used with Mexican patients with colorectal cancer. The inclusion criteria were having a diagnosis of colorectal cancer, being over 18 years of age, and knowing how to read and write. Patients with a previous history of cancer or psychiatric disorders were excluded.

### Questionnaires

#### Sociodemographic and medical data questionnaire

The questionnaire included sociodemographic data such as age, marital status, work, schooling, and monthly income. Clinical data included type of cancer, clinical stage, and type of treatment.

#### Impact of Event Scale-Revised (IES-R)

The IES-R was designed by Horowitz et al. (1979) to assess subjective discomfort caused by stressful and/or traumatic experiences. It is one of the most commonly used instruments for the measurement of post-traumatic symptoms in adults (Ferrer & Delgado, 2018). The Spanish version by Caamaño et al. (2011) is based on a review of the IES-R by Weiss and Marmar (1997) that utilized the diagnostic criteria of the DSM-IV and consists of 22 items grouped into three subscales: intrusive thoughts, avoidance, and hyperarousal. It had a total Cronbach’s alpha of 0.98. Cronbach’s alpha for our study’s population was 0.91.

#### Hospital Anxiety and Depression Scale (HADS)

Designed as an instrument to detect states of anxiety and depression in the hospital environment, HADS is one of the most widely used instruments in oncology for measuring distress (Linden et al., 2012). The Spanish version, which has been used for Mexican cancer patients, has 12 items divided into two subscales — depression and anxiety; they have a total Cronbach’s alpha of 0.86 (Galindo et al., 2015). Cronbach’s alpha for our study was 0.72. The HADS has good psychometric properties for discriminating between cancer patients with and without the presence of clinical distress (López et al., 2012).

#### Life Orientation Test (LOT-R)

The LOT-R was designed by Scheier et al. (1994) to measure the degree to which people generally have favorable expectations of their future. Likewise, the Spanish version is an adequate and practical tool for diagnostic purposes and epidemiological research in samples from Latin America (Zenger et al., 2013). We used the Spanish version; it is comprised of 10 items and has a reliability of α = 0.79 (Otero et al., 1998). Cronbach’s alpha for our study was 0.57.

### Procedure

The patients were recruited from a public hospital on the basis of a review of medical files. Once the review identified the patients who met the inclusion criteria, they were searched out in the waiting room and invited to participate. Subsequently, they were asked about previous or current diagnoses of cancer and mental illness to identify whether they met the inclusion criteria. Then, the purpose of the research, the confidentiality of the data provided, and their right to receive information about their doubts regarding the study were explained to them. Once they decided to participate in the study, the participants signed an informed consent form. Subsequently, the questionnaires on symptoms of post-traumatic stress, distress, and optimism were administered. Participants did not receive any type of reward for their participation.

The data were analyzed using SPSS 24. Descriptive data of frequency and percentages were obtained for categorical variables, and means and standard deviations for continuous variables. For the prevalence of post-traumatic stress symptoms, the cut-off point of ≥ 20 suggested by Costa and Gil (2007) was used; the cut-off point of ≥ 10 suggested by Costa et al. (2009) was used for distress. The Kolmogorov-Smirnov normality test was performed, in which p values <.01 were obtained, which indicated non-normality. Therefore, non-parametric tests were used to analyze the relationship between the psychosocial, sociodemographic, and medical variables. The Mann-Whitney U test was used for variables with two categories and the Kruskal-Wallis test was used for variables with more than three categories; Spearman’s correlation was used for continuous variables. The Holm-Bonferroni correctness test was performed to prevent the type I error.

Effect size was obtained using Cohen’s d to measure the relative strength of the differences between the means of two groups (d). The values indicated by Cohen (1988) are the following: d = 0.20 to 0.49 is considered low effect, d = 0.50 to 0.79 a medium size effect, and d = 0.80 or greater a large effect. Likewise, the partial square eta was used to obtain the effect size of three or more groups (η^2^p). The values indicated by Cohen are the following: η^2^ = 0.01 is considered low effect, η^2^ = 0.06 a medium size effect, and η^2^ = 0.14 a large effect.

## Results

The data collection period was from March 2016 to August 2017. A total of 206 patients was evaluated, of which 14 submitted incomplete questionnaires; therefore, the data of 192 patients with colorectal cancer were analyzed. Their characteristics are described in *Table 1*. The age range of the participants was from 22 to 82; most of the participants were female, not currently working, and married. Regarding the prevalence of post-traumatic stress symptoms, 32.3% presented clinically significant symptoms. Further, 21.4% of the patients presented clinically significant emotional distress. Likewise, significant differences were found between men and women in levels of post-traumatic stress and distress, with medium effect size for post-traumatic stress.

**Table 1:** Prevalence and comparison analysis between psychosocial, sociodemographic and medical variables (N = 192)

	n	%	EIE–R	HADS M(SD)	LOT–R
Sex					
Female	102	53.1	21.9(16.5)	4.8(3.9)	40.1(4.3)
Male	90	46.9	13.6(11.7)	7.5(4.9)	38.7(5.6)
P value			.000**	.000**	.129
d			.580	–.609	.280
Works					
Yes	56	29.2	17.0(14.2)	5.0(4.1)	40.3(4.6)
No	132	68.8	17.8(15.2)	6.6(4.7)	39.1(5.1)
P value			.906	.024*	.158
d			–.054	–.362	.247
Marital status					
Married	137	71.4	17.7(14.3)	6.1(4.5)	39.8(5.1)
Single	22	11.5	15.4(16.0)	7.0(5.2)	38.4(4.7)
Divorced	18	9.4	20.2(18.1)	5.5(4.7)	38.8(5.5)
Widower	14	7.3	15.5(13.9)	5.5(4.1)	39.2(3.4)
P value			.407	.807	.423
η^2^			.007	.007	.010
Type of cancer					
Colon cancer	96	50.0	16.9(15.5)	6.1(5.0)	39.9(4.8)
Rectal cancer	96	50.0	18.1(13.9)	6.0(4.1)	39.0(5.2)
P value			.296	.688	.165
d			–.081	.021	.179
Clinical stage					
I	15	7.8	17.8(14.4)	6.1(8.0)	41.1(3.8)
II	53	27.6	16.2(16.7)	5.9(4.7)	38.8(5.3)
III	86	44.8	17.5(14.4)	5.9(4.0)	39.5(4.9)
IV	30	15.6	17.4(13.1)	6.0(3.8)	39.8(5.3)
P value			.609	.763	.546
η^2^			.003	.002	.012
Surgery					
Yes	155	80.7	21.7(13.8)	5.8(4.7)	39.7(4.7)
No	37	19.3	16.5(14.8)	7.1(4.0)	38.4(5.9)
P value			.013*	.039*	.154
d			.363	–.297	.243
Colostomy					
Yes	105	54.7	17.5(14.2)	5.9(4.3)	39.5(4.7)
No	87	45.3	17.6(15.4)	6.2(4.9)	39.5(5.3)
P value			.719	.710	.739
d			–.006	–.065	0
Current treatment					
RT / QT Neo	23	12.0	26.0(16.1)	8.1(4.1)	38.5(6.5)
RT or QT Ady	56	29.2	15.8(14.7)	6.1(4.6)	38.9(4.8)
Palliative QT	50	26.0	17.0(13.4)	6.2(3.8)	38.3(5.3)
Observation	59	30.7	16.1(14.4)	5.1(5.2)	41.3(3.8)
P value			.054	.022*	.015*
η^2^			.048	.037	.065

Note. Continuous mean (SD) categorical %; ** p ≤ .001 Alpha adjusted by Holm Bonferroni test * p ≤ .05; SD = Standard Deviation. d = Cohen´s d. η= partial square eta. IES-R = Impact of Event Scale — Revised. ^2^ HADS = Hospital Anxiety and Depression Scale. LOT-R = Life Orientation Test. RT / QT Neo = Neoadjuvant Radiotherapy / Chemotherapy. RT or QT Ady = Radiotherapy or Adjuvant Chemotherapy. Palliative QT = Palliative chemotherapy.

Post-traumatic stress was different between patients with and without surgery, with low effect size. For distress, differences were found with significant trends for patients with and without surgery, with and without work, and type of current treatment with low effect size. As for optimism, tendencies for significance were only found in the type of current treatment, with medium effect size. No significant differences were found for the other comparisons (*Table 1*).

*Table 2* shows that post-traumatic stress and distress symptoms were negatively and significantly related to age, monthly income, and optimism. Post-traumatic stress symptoms were positively and significantly related to distress. Optimism was positively related to monthly income.

**Table 2:** Correlation analysis between psychosocial and sociodemographic variables (N = 192)

	M(DE)	IES–R	HADS rs	LOT–R
Age	54(12.6)	–.262**	–.277**	–.004
Monthly income	7742.7(7350.9)	–.258**	–.247**	.258**
Scholarship	9.6(4.2)	–.090	–.099	.044
IES-R			.657**	–.273**
HADS				–.450**

Note. ** p≤.01 * p≤.05. M = Mean. SD = Standard Deviation. r_s_ = Spearman’s Correlation. EIE-R = Revised Event Impact Scale. HADS = Hospital Anxiety and Depression Scale. LOT-R = Life Orientation Test.

## Discussion

The general objective of this study was to evaluate the levels of symptoms of post-traumatic stress, distress, and optimism, as well as to examine their differences relative to sociodemographic and medical variables in Mexican patients with colorectal cancer.

In our study, 32.3% of patients with colorectal cancer presented symptoms of post-traumatic stress. These results are similar to those of Naidich and Motta (2000), who found that women with breast cancer had a current incidence of post-traumatic stress symptoms of 32%, which was significantly higher than that in women without cancer.

However, the prevalence of post-traumatic stress found in this study was lower than that reported in veterans with colorectal, gastric, esophageal, and head and neck cancer, of whom 85.5% reported having experienced post-traumatic stress symptoms related to their experience of cancer. These differences may have occurred because that study used a different instrument than the one in our research, and because participants were military veterans diagnosed with cancer. Combat-related post-traumatic stress disorder has been found to increase the risk of developing post-traumatic stress symptoms related to cancer diagnosis and treatment (Schuster et al., 2014). By contrast, the present investigation showed a higher prevalence of post-traumatic stress than that for patients with breast cancer after surgery (18.5%) and at their six month follow-up (16.3%) (Mehnert & Koch, 2007).

Regarding distress, 21.4% of patients presented symptoms of clinically significant emotional distress. These results are low compared to those of other investigations, such as the longitudinal study by Dunn et al. (2013), where clinically significant levels of psychological distress were reported in up to 49% of 1703 colorectal cancer patients five months after diagnosis. However, our results are high compared to those of a study that was conducted through an Australian prospective survey of 1822 patients with colorectal cancer; that study reported significant levels of psychological distress in 8.3% and 6.7% of patients at 6 and 12 months after diagnosis, respectively (Lynch et al., 2008). By contrast, in our study, it was observed that the levels of symptoms of post-traumatic stress and distressaccording to sociodemographic and medical variables.

In relation to post-traumatic stress symptoms, a significant negative relationship was obtained depending on age and monthly income. These results are similar to those of Schuster et al. (2014), who showed in a sample of veterans with colorectal cancer that younger age was associated with more symptoms of post-traumatic stress related to cancer. This may be because younger people are generally more distressed after being diagnosed with cancer than older patients (Kangas et al., 2002). In addition, they are probably less used to receiving diagnoses of this magnitude, which they may perceive as traumatic (Pereira et al., 2012).

On the other hand, O’Connor et al. (2011) reported that low social status was one of the predictors of severe post-traumatic stress symptoms 15 months after breast cancer surgery. Furthermore, in this study, women had more post-traumatic stress symptoms than men, with medium effect size. These results are similar to those of Rucklos and Frombach (2000), who observed that in cancer patients with a diagnosis at least 12 months old, women reported symptoms of post-traumatic stress more frequently. Current studies report that cancer patients may suffer from financial distress during and after the cancer process; this distress is usually determined by sociodemographic, financial, and employment factors (Pauge, et al., 2021; Semin et al., 2020).

In our research, significant differences in post-traumatic stress symptoms were found between patients with and without surgery, with low effect size. These results are similar to those reported in patients with breast cancer where 20.1% of women had total scores suggesting severe post-traumatic stress symptoms three months after surgery (O’Connor et al., 2011). Likewise, colorectal cancer patients who underwent surgery/chemotherapy or surgery/radiotherapy had more symptoms of post-traumatic stress disorder than those who didn’t (Pereira et al. 2012). These results may be due to side effects after surgery, such as diarrhea and sexual dysfunction (Benedict et al., 2018; Milbury et al., 2013). They may also have occurred because in some patients who undergo surgery, it is necessary to create a stoma, which, depending on the extent of the disease and the surgical procedure, can be temporary or permanent (Sharpe et al., 2011). However, this study did not find differences between patients with and without a colostomy, so it would be interesting to inquire about what specific aspects of surgery are associated with post-traumatic stress in patients with colorectal cancer.

A significant and negative relationship was found between distress, and age and income. Likewise, higher distress scores were found in men than women, as well as in patients who did not work, those who did not undergo surgery, and patients undergoing radiation treatment and/or neoadjuvant chemotherapy, with low effect size. This is similar to what was reported in a study of colorectal cancer survivors in which young men, in a late stage of the disease, with low educational levels and social support, were more likely to have a high level of distress (Dunn et al., 2013). Therefore, it is considered important to inquire about cancer-related concerns in younger adults. Additionally, our results are similar to those of Miles et al. (2017) that suggested that age is a significant predictor of distress related to colorectal cancer. Some authors explain that the negative associations between age and distress may be due to the unexpectedness of the diagnosis and a greater impact on the life of the patient and their family members, as well as on work commitments (Green et al., 1998).

Iconomou et al. (2004) showed in their study, which sampled outpatients with colorectal, genitourinary, lung, and breast cancer, that almost a third of patients experienced severe psychological distress during chemotherapy. Cancer patients who receive chemotherapy or radiotherapy may perceive the cancer as more serious, may have less control over the treatments, and therefore show higher symptoms of psychological distress. Likewise, more frequent trips to the hospital and longer periods of treatment can contribute to greater psychological comorbidity (Denlinger & Barsevick, 2009; Pereira et al., 2012).

This result is consistent with the finding of Baker et al. (2005) who showed that patients who were currently undergoing cancer treatment reported, on average, a significantly higher number of problems related to the disease, compared to those who were not currently undergoing treatment. These data were obtained by patients diagnosed with lung cancer, followed by survivors of breast, colorectal, and prostate cancer. Pettersson et al. (2014) report that 6% of colorectal cancer patients receiving chemotherapy score high (quite or very much) on the dimension of distress related to the problem, which indicates that patients experience symptoms of psychological distress both at the beginning and during the treatment phase.

As to the association between the psychological variables, our results show that post-traumatic stress symptoms are positively and significantly related to distress, which is similar to the finding by Salsman et al. (2009) that post-traumatic stress disorder symptomatology is positively associated with depression and anxiety. In addition, symptoms of post-traumatic stress are associated with depression and anxiety symptoms in cancer patients admitted for unplanned hospitalization (Nipp et al., 2019).

Regarding optimism, a positive and significant relationship was obtained with monthly income and a significant difference depending on the current stage of treatment, with medium effect size. That is, patients under observation showed the highest optimism score. This is consistent with the findings by Deimling et al. (2006) that long-term cancer survivors were less optimistic in the time closest to cancer diagnosis, which may be due to symptoms of cancer and its treatment. Likewise, Cro (2014) observed that self-perception of good or excellent health was significantly associated with higher optimism in long-term survivors of breast cancer. These results are consistent with those of our study because the patients were not undergoing cancer treatment.

In this regard, Taber et al. (2016) found that optimism may be associated with beneficial health-related outcomes among cancer survivors. Additionally, analyses by Meng et al. (2012) showed that low optimism is a risk factor for increased distress in colorectal cancer survivors. Similarly, the results of Applebaum, Stein et al. (2014) showed that greater optimism is significantly associated with fewer anxiety and depression symptoms in patients with advanced cancer. This highlights the importance of optimism in facilitating psychological adaptation to living with cancer.

Thus, the previous results showed the importance of psychosocial factors in cancer diseases such as post-traumatic stress symptoms, distress, and optimism. In this regard, Schuster et al. (2014) mentioned that patients’ individual and psychosocial characteristics can play an important role in variables related to the disease, and help determine an individual’s response to the stress of cancer. Similarly, in line with Kangas et al. (2002), future research needs to identify the influence of different stressors that can occur during cancer experience. In addition, Pereira et al. (2012) suggested that health professionals should also be attentive to signs of clinical distress and have a better understanding of the factors that can predict cancer-specific distress, which could allow for better clinical and psychoeducational interventions.

## Conclusion

A significant percentage of colorectal cancer patients present high levels of post-traumatic stress symptoms as well as distress, and these levels may vary according to sociodemographic and medical characteristics.

## Limitations

This study had some limitations. First, the Cronbach’s alpha of the optimism scale was low for this study so the results should be interpreted with caution. Also, a descriptive cross-sectional design does not allow drawing inferences about the direction of causality. In this sense, we recommend carrying out longitudinal studies that would allow us to observe the trajectory of the psychosocial variables that patients present over time. Likewise, given that the results are only from patients found at a hospital entity, they cannot be generalized. Thus, future research is required to confirm these findings.

Nevertheless, this study is valuable because it shows the prevalence of symptoms of post-traumatic stress and considerable distress in Mexican patients with colorectal cancer, and how these symptoms differ according to sociodemographic and medical conditions. This highlights the need to provide care to these patients so that they can effectively manage the consequences of the disease. With this information, health professionals could identify the patients most likely to experience distress and refer them to psycho-oncology services. In accordance with the above, this study served to promote more comprehensive work in oncology services.
